# Exploring the Gut Microbiome Alteration of the European Hare (*Lepus europaeus*) after Short-Term Diet Modifications

**DOI:** 10.3390/biology10020148

**Published:** 2021-02-13

**Authors:** Anna Padula, Marina Bambi, Chiara Mengoni, Claudia Greco, Nadia Mucci, Ilaria Greco, Alberto Masoni, Sara Del Duca, Giovanni Bacci, Giacomo Santini, Renato Fani, Marco Zaccaroni

**Affiliations:** 1 Institute for Environmental Protection and Research, Conservation Genetics Area, Via Ca’ Fornacetta 9, Ozzano dell’Emilia, 40069 Bologna, Italy; chiara.mengoni@isprambiente.it (C.M.); claudia.greco@isprambiente.it (C.G.); nadia.mucci@isprambiente.it (N.M.); 2 Department of Biology, University of Florence, Via Madonna del Piano 6, Sesto Fiorentino, 50019 Florence, Italy; marina.bambi@unifi.it (M.B.); ilaria.greco@unifi.it (I.G.); alberto.masoni@unifi.it (A.M.); sara.delduca@unifi.it (S.D.D.); giovanni.bacci@unifi.it (G.B.); giacomo.santini@unifi.it (G.S.); renato.fani@unifi.it (R.F.); marco.zaccaroni@unifi.it (M.Z.)

**Keywords:** *Lepus europaeus*, gut microbiota, diet modification, faecal samples, hindgut fermenters

## Abstract

**Simple Summary:**

The composition of gut microbial communities can be altered by host diet shift. In this study, we investigated the microbiome composition of European hares and the potential changes in their gut communities after 4 days from the introduction in the diet of new nourishment. The control group was fed with standard fodder; the diet of the experimental group was integrated with apples and carrots. DNA was extracted from fresh faecal pellets and the *V3-V4* hypervariable regions were amplified and sequenced using the Illumina MiSeq^®^ platform. The amplicon sequence variants were classified into 735 bacterial genera belonging to 285 families and 36 phyla; the most abundant phyla represented by *Bacteroidetes* and *Firmicutes.* Experimental and control hares did not show statistically significant differences in their microbial communities suggesting the exposition time to a new diet should be extended to define the time frame necessary to affect microbiome composition.

**Abstract:**

This study aimed to characterise the gut microbiome composition of European hares (*Lepus europaeus*) and its potential changes after a short-term diet modification. The high sensitivity of European hare to habitat changes makes this species a good model to analyse possible alterations in gut microbiome after the introduction of additional nourishment into the diet. In total, 20 pairs were chosen for the experiments; 10 pairs formed the control group and were fed with standard fodder. The other 10 pairs represented the experimental group, whose diet was integrated with apples and carrots. The DNA from fresh faecal pellets collected after 4 days from the start of the experiment was extracted and the *V3-V4* hypervariable regions were amplified and sequenced using the Illumina MiSeq^®^ platform. The obtained amplicon sequence variants were classified into 735 bacterial genera belonging to 285 families and 36 phyla. The control and the experimental groups appeared to have a homogenous dispersion for the two taxonomic levels analysed with the most abundant phyla represented by *Bacteroidetes* and *Firmicutes.* No difference between control and experimental samples was detected, suggesting that the short-term variation in food availability did not alter the hares’ gut microbiome. Further research is needed to estimate significant time threshold.

## 1. Introduction

Climate change, pollution, and loss of suitable habitat are considered the main causes of small mammals decline [1]. Agricultural intensification plays a major role in habitat loss: the shift from small cultivated patches to extensive cereal crops reduces the presence of spontaneous vegetation, impacting the diet of mammals that rely on a variety of seasonal food to match their energetic requirements [2,3].

When food requirements do not meet resource availability, individuals can be more sensitive to diet modifications that can lead to microbiota gut alteration and thus to gastrointestinal diseases [4], pointing out a tight correlation between the host’s wellbeing and microbiota composition [5]. The complex interactions existing between microbial communities and their hosts are driven by a multitude of environmental factors such as age, body condition, genetics, lifestyle, climate, and host’s diet [5,6], and can affect the microbiota composition throughout their life. In particular, several studies highlighted how diet could determine changes in the gut microbiota [5,7,8,9,10]. The investigations of the gut microbial communities can successfully be performed analysing DNA extracted from faeces, and they are widely carried out for several applications and researches [11,12]. Progresses in high-throughput sequencing technology and bioinformatic techniques allow accurate analysis of the fluctuations of the gut microbial composition throughout the host lifespan, usually focusing on bacteria [13].

Gut microbiome composition is commonly inferred from 16S rRNA gene sequence, a component of the 30S small subunit of the bacterial ribosome, characterised by variable (V1-V9) and highly conserved regions suitable for primer binding [14] whose characterisation allows the taxonomic assignment of the microorganisms. Mammalian microbiome research has a long history [15], marked by increases in scale and scope due to next-generation sequencing technologies (NGS) and in associated computational methods.

The European hare (*Lepus europaeus*) is both a game and prey species, worldwide distributed. In Europe, many populations record high densities, although they have experienced a severe decline since the 1960s [1]. As a consequence of this contraction, the species was listed in Appendix III of the Bern Convention on the European Wildlife and Natural Habitats and is still classified as “threatened” or “endangered” in several countries [1,16,17], although it has been flagged as “least concern” by the International Union for Conservation of Nature (IUCN, red list 2017).

Different from other leporids, European hares face higher energetic costs due to life habits [18].

Hares feed on plants rich in polyunsaturated fatty acids and avoid crude fibres [19], especially during summer when winter reserves have been exhausted, and females have to nurse young cubs [6]. Particularly, hare seems to prefer crops during the breeding season (from January to October), spontaneous herbs in spring-summer [16], graminoids and cereals in autumn-winter [20].This choice could be related to seasonal energy requirements. As for other small mammals, lagomorphs are highly influenced by diet change, and the main critical consequence is the modification or alteration of gut microbiota [4].

The limited literature about microbiome communities in hares and the species particular sensitivity to diet modifications makes the European hare an eligible target species to analyse possible microbiota changes after the introduction of new nourishment into its diet.

The gut microbiome can be rapidly and strongly altered by host diet shift, particularly when the fibres intake changes [21]. In small mammals, shifts in gut microbial communities could appear after 24 h from the introduction in the diet of new types of food [22,23], but longer times have been described as well [24], suggesting considerable interspecific variability [21]. In this study, we aimed to investigate the microbiome composition of hares and the potential changes in their gut communities after a short-term diet modification. Aiming to identify conditions and clear time required for evidence of changing, we designed a study under controlled conditions (time and diet), thus analysing the microbiome composition in twenty pairs of hares, half-fed with a standard diet (control group), and a half following a modified diet (experimental group). We established 4 days as the exposition time, according to [25].

This preliminary analysis might be useful for future research on free-living hares with significant implications for the conservation and management of this species [26,27].

## 2. Materials and Methods

### 2.1. Samples Collection

To assess microbiome composition under controlled conditions, we used 40 adult European hares (20 males and 20 females) paired in 20 reproductive enclosures, and representing the fourth bred generations. In total, 10 pairs were randomly assigned to the control group, and the remaining 10 used as the experimental group. The study was carried out in a game farm (Centro Pubblico Produzione Selvaggina C.P.P.S. Montalto, www.cppsmontalto.it accessed on 31 August 2020, in the *province of Grosseto*, Tuscany, Italy).

The two groups were fed with two different diets. The control group was fed with fodder, which constitutes the hare’s regular diet composed by alfalfa (*Medicago sativa*), wheat (*Triticum* sp.), sunflower (*Helianthus* sp.) seeds, oat (*Avena sativa*), sugar beet (*Beta vulgaris*), grass flour, sugar cane (*Saccharum* sp.), cocoa husks (*Theobroma cacao*), palm (*Elaeis guineensis*) and soy (*Glycine max*) oil. In the experimental group, the standard diet was integrated with apples (*Malus domestica*) and carrots (*Daucus carota*) available *ad libitum*, which were never used to feed the hares before and which increase sugar (digestible carbohydrates) and fibre (no-digestible carbohydrates) levels in the diet. For each enclosure, fresh faecal pellets were collected after 4days of controlled diet and within 10 h from defecating. Samples were preserved into empty sterile 50 mLFalcon^®^ tubes, containing both male and female scats belonging to the same enclosure, and stored at −20 °C until processed.

### 2.2. DNA Extraction and Amplification

The faecal DNA was extracted using the QIAamp DNA Stool Mini Kit (QIAGEN GmbH, Valencia, CA, USA), primarily used for the extraction and purification of DNA from fresh/frozen scats and degraded DNA, which also allows the removal of PCR inhibitors. The DNA extracted was used to create PCR amplicons libraries, using the 341F (5′-CCTACGGGNBGCASCAG-3′) and 805R (5′-GACTACNVGGGTATCTAATCC-3′) primers specific for the *V3-V4* hypervariable regions and adding multiplexing indices and Illumina sequencing adapters. Libraries have been normalised and sequenced on the Illumina MiSeq^®^ platform (Illumina, Inc., San Diego, CA, USA) with a 2 × 300 bp paired-end run. During the preparation of the libraries for sequencing, internal positive and negative controls were used to check for contamination and correct sequencing execution. PCR amplification, library construction, and sequencing were performed by an external company (IGA Technology Services, Udine, Italy). Sequence files were deposited in the NCBI sequence read archive (SRA) under the accession PRJNA627685.

### 2.3. Amplicon Sequence Variants Detection

Sequences were clustered into Amplicon Sequence Variants (ASVs) using the DADA2 pipeline (https://benjjneb.github.io/dada2/tutorial.html, accessed on 31 August 2020) [28,29] through the R software version 3.4.3 [30]. Primers used for PCR amplifications were removed with “cutadapt” [31] using default settings. Sequences containing no primers were removed (“discard-untrimmed” option). When the adapter was found only in one mate, both pairs were discarded to maintain paired-end structure (“pair-filter = any” option). Sequences were filtered with the “filterAndTrim” function of DADA2 using a maximum error rate of 2. Reads were chopped at 270 bp (forward) and 200 (reverse) with the “truncLen” option to maintain more than 20 bp of overlap while removing low-quality tails. Sequences were denoised and merged, and variants were inferred using the DADA2 algorithm. Taxonomic annotation was carried out after chimera removal with the Silva training set 132 [32].

### 2.4. Statistical Analysis

The microbiome composition among samples was investigated using multivariate techniques. Before doing the analysis, microbial communities’ richness was checked through the rarefaction curves on the ASVs assignment. The multivariate distances among samples were computed with the Bray—Curtis dissimilarity index after log-transforming data (to reduce the asymmetry in Taxa distributions) and the resulting distance matrix was analysed by non-Metric Multidimensional Scaling (nMDS) according to [33]. Differences in bacterial composition were tested with a permutation-based non-parametric multivariate analysis of variance (npMANOVA), as described in [34], using the factor “Diet” (fixed). Microbial diversity among samples was also tested with a multivariate dispersion analysis following [35]. All the analyses were carried out with the R software v.3.6 [36] using “vegan” [37] and “phyloseq” [38] packages.

## 3. Results

**Figure 1:**
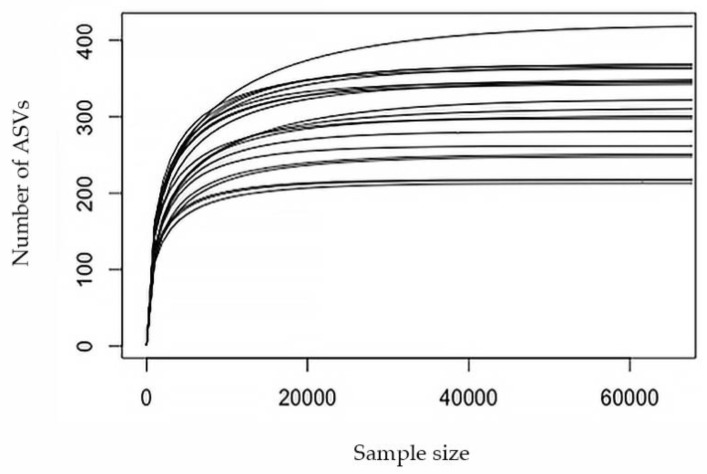
Rarefaction curves based on the number of reads (sample size) and the number of ASVs in each sample. Each line represents one sample. No significant differences were recorded.

Bacterial communities of faecal samples examined through NGS analysis showed a sequencing yield of 2,566,753 paired sequences. The 61.63% of the initial pairs were correctly merged (1,881,061 sequences) with a mean of 94,053 sequences *per* sample. Quality filtering steps produced 1,974,276 high-quality sequences that were correctly mapped into 17,108 ASVs. The removal of chimaeras produced 1,582,138 total sequences with an average of 7500 ASVs *per* sample. Representative sequences for each ASV were correctly classified into 735 bacterial genera belonging to 285 family and 36 phyla. Furthermore, all the rarefaction curves reached the plateau suggesting a good representation of the microbial community for all samples (Figure 1).

**Figure 2:**
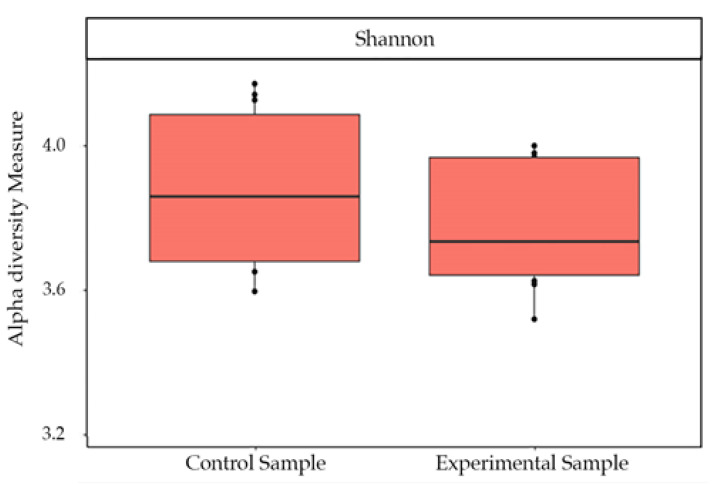
Shannon diversity calculated on raw data. Box plots show the indices based on samples bacterial communities in control and experimental samples. No significant differences were recorded.

The Shannon index of bacterial communities measured on the number of ASVs detected did not show a difference in the diversity in the two different conditions (*p* value = 0.91) (Figure 2).

**Figure 3:**
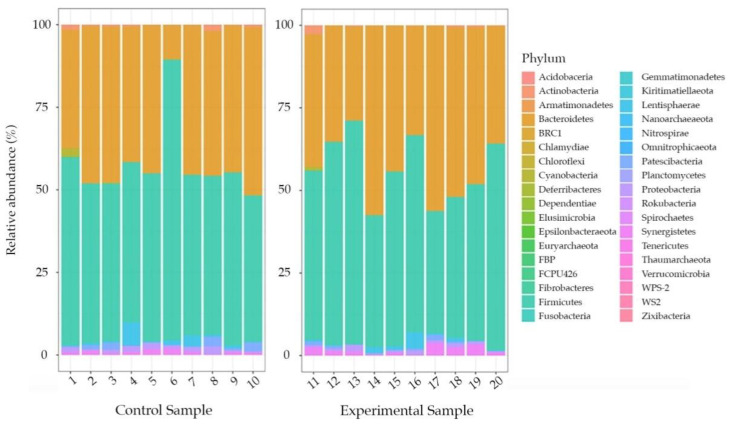
Bar plots showing the relative abundances of bacterial phyla in the control and experimental samples. We reported the ASVs > 3% of the whole community. The two groups showed similar bacterial composition.

Control and experimental groups resulted to have a relatively homogenous dispersion of phyla (npMANOVA: F_1,18_ = 0.55, *p* = 0.64) and their relative abundance did not exhibit substantial variations among samples (Figure 3).

In both cases, the most abundant phyla were *Bacteroidetes* and *Firmicutes* that represented on average (±SEM) the 40% (±2.7) and 50% (±3), respectively, of the ASVs detected in control samples. The only exception was represented by sample #6, which deviates from this trend since *Firmicutes* and *Bacteroidetes* comprise the 84.9% and 10.3% of the ASVs detected, respectively (see supplementary: Tables S1 and S2).

**Figure 4:**
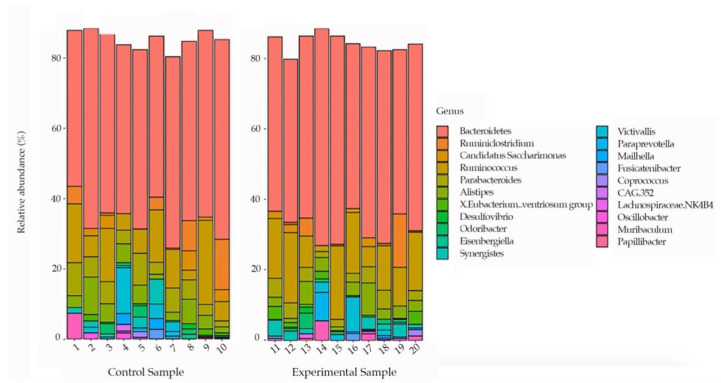
Bar plots showing the 10most abundant bacterial genera. We represented the ASVs > 1% among the 10most abundant bacterial genera. Each column represents a sample.

Comparing samples at the genus level, *Bacteroides* were the most represented taxonomic group, with approximately 50% in all samples (Figure 4). In particular, in Figure 4 we represented the ASVs > 1% among the 10most abundant bacterial genera.

**Figure 5:**
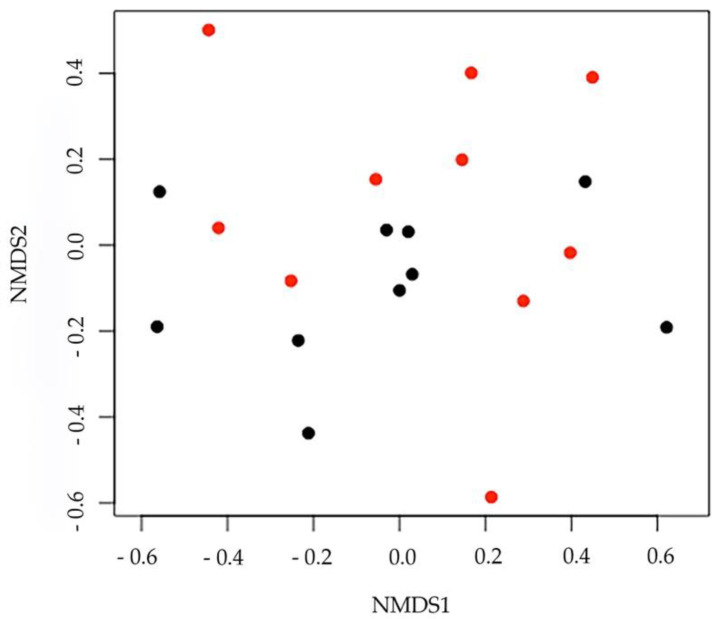
Results of nMDS at the genus level. Controls and experimental samples are represented by red and black dots, respectively.

Data concerning the nMDS at the genus level are shown in Figure 5. The stress levels were 0.18 (for the phylum) and 0.16 (for the genus), suggesting that the ordination plot adequately represented multivariate distances among samples. Additionally, at this taxonomic level, samples did not show significant variability between control and experimental diet (npMANOVA: F_1,18_ = 0.38, *p* = 0.67). Data obtained confirmed the lack of difference between experimental and control samples, as the data points of the two groups are completely overlapped with no clear separation.

## 4. Discussion

Lagomorphs are known to be sensitive to habitat change and food availability, with sudden diet modifications that can cause a shift in their microbial community and a consequent higher risk of gastrointestinal diseases [39,40]. Furthermore, the lack of literature about the microbial communities of hares makes bred hares a perfect target for a pilot study on this topic. In the present study, we aimed to detect the eventual modification of the gut hare microbiota after the exposure to new nourishments.

The bacterial communities of the control (fed with fodder) and the experimental group (fed adding carrots and apples to fodder diet) were both dominated by *Firmicutes* and *Bacteroidetes* phyla, which corresponded approximately to 90% of the detected ASVs. These results are consistent with previous researches based on a variety of mammalian gut studies [1,41]. Data showed high similarity with microbial communities found in several herbivorous ruminant mammals [42] and are comparable to results obtained in other studies carried out on wild hares and other species, as beaver and rabbit characterised by a hindgut fermentation [43,44]. The presence of these phyla was also found in the core rumen microbiome and the enlarged crop of Hoatzin (*Opisthocomus hoazin*), a unique folivorous bird, suggesting their essential role in the fermentation processes and lignocellulose digestion. Furthermore, previous studies on other species (Hoatzin and cows) highlighted a positive correlation between similar diets and analogous microbial communities [45,46,47]. Data obtained for hares reared under controlled conditions and subjected to a strict diet comply with the information found in the literature for wild hares [6] and highlighted the presence of similar gut microbial communities.

On the other hand, the results did not show any statistically significant variation in the microbial composition after the diet alteration in the experimental groups at the phylum level. Although the two groups of hares exhibited a similar microbial community in terms of phyla, the experimental group might seem to display a greater abundance of the two main phyla (*Bacteroidetes* and *Firmicutes*) compared to the control group. Moreover, even the most abundant genera found in each sample did not present a statistically significant variation, showing a similar microbial composition where *Bacteroides* represents the main abundant taxonomic group in both conditions.

The slight variation may suggest different scenarios: first, we could interpret it as the beginning of a change of the gut microbial community; hence, it cannot be excluded that a prolonged diet might be more effective in causing a stronger alteration of microbial gut composition. However, gut microbiome could display transient alterations due to a short-time diet modification, particularly in the first 24-h, then reverting back to its pre-intervention state [48]. So, our results may suggest a different scenario, and we may have taken a too long time to record the effects of the food change.

Unlike the sensitivity to trophic resources alteration found for some small mammal species that can be affected by gut microbiome alteration already after 24-h [22,23] of changes in diet, the hare microbiome seemed to have a higher tolerance range to food modification at least in the experimental conditions used in this work.

Exposition to enriched diet was very likely too short to allow a significant modification of microbial composition in the experimental group. Long-term diet based on regular fodder, instead, seemed to have a major impact also on the experimental group, since the same microbial community composition was found despite addition of new nutrients.

Although gut microbiome changes can be detected within 24 h after diet modification, only a long-term diet has been associated with a stable diversification of gut microbiome composition [49]. This can confirm the powerful effect of the diet on the gut microbiome when the host organism is exposed for a prolonged time at the same diet [22].

In conclusion, experimental and control hares did not show statistically significant differences in their microbial communities, both dominated by *Bacteroidetes* and *Firmicutes*. Data obtained in this work strongly suggest that, despite the particular sensitivity to microbiome changes [4,50], the exposition time to a new diet should be replicated and extended to define the minimal time frame necessary to affect microbiome composition. In addition to this, bred and wild hares share a similar composition of microbiomes.

This study opens the possibility to future comparison between gut microbiome in different leporids, especially of management and conservation concern as *Lepus corsicanus*.

## Supplementary Materials

The following are available online at https://www.mdpi.com/2079-7737/10/2/148/s1. Table S1: Table showing the percentage of the most abundant bacteria phyla in control samples. Table S2: Table showing the percentage of the most abundant bacteria phyla in experimental samples.
