# Supplementary material for: Exploring the Gut Microbiome Alteration of the European Hare (Lepus europaeus) after Short-Term Diet Modifications
Source: Biology (Basel). 2021 Feb 13;10(2):148. doi: 10.3390/biology10020148 (PMC7918456; doi:10.3390/biology10020148)
Supplement: Supplementary file 1 [file biology-10-00148-s001.pdf]

Table S1. Table showing the percentage of the most abundant bacteria phyla in control samples.

	Control Samples								
**Sample ID**	**Firmicutes**	**Bacteroidetes**	**Proteobacteria**	**Synergistetes**	**Lentisphaerae**	**Actinobacteria**	**Patescibacteria**	**Tenericutes**	**Cyanobacteria**
1	48.7	45.2	1.4	0.9	3.2	0.1	0.2	0.0	0.0
2	56.9	35.8	1.4	0.8	0.2	1.4	0.3	0.0	2.7
3	48.1	43.9	2.4	0.1	0.5	1.8	3.0	0.0	0.0
4	48.3	47.6	0.3	1.2	0.6	0.4	1.2	0.1	0.0
5	48.6	41.0	1.8	0.9	6.9	0.5	0.0	0.0	0.0
6	50.9	44.8	1.9	1.7	0.2	0.1	0.0	0.0	0.0
7	52.3	44.5	0.5	0.8	0.9	0.1	0.5	0.0	0.0
8	47.9	47.3	0.9	0.5	0.3	0.5	2.1	0.1	0.0
9	84.9	10.3	0.1	1.9	1.4	0.0	0.0	0.0	0.0
10	44.2	50.8	0.2	0.7	0.3	0.7	2.7	0.0	0.0

Table S2. Table showing the percentage of the most abundant bacteria phyla in experimental samples.

	Experimental Samples.								
**Sample ID**	**Firmicutes**	**Bacteroidetes**	**Proteobacteria**	**Synergistetes**	**Lentisphaerae**	**Actinobacteria**	**Patescibacteria**	**Tenericutes**	**Cyanobacteria**
11	52.8	44.1	0.6	0.8	1.3	0.1	0.2	0.0	0.0
12	47.5	47.3	0.9	3.3	0.1	0.6	0.0	0.0	0.0
13	67.8	28.4	2.1	0.8	0.1	0.3	0.0	0.3	0.0
14	59.7	33.0	1.3	0.2	4.8	0.1	0.6	0.0	0.0
15	62.6	35.6	0.2	1.0	0.3	0.1	0.0	0.0	0.0
16	37.0	56.1	0.6	3.8	0.4	0.1	1.6	0.0	0.0
17	51.2	40.1	0.6	2.4	0.5	2.6	1.1	0.0	1.1
18	39.8	57.4	0.4	0.2	1.9	0.0	0.0	0.0	0.0
19	42.5	51.4	1.3	2.4	1.2	0.5	0.4	0.0	0.0
20	61.6	35.1	0.6	1.2	0.1	0.1	0.9	0.1	0.0
